# Diabetes mellitus is associated with 90-day mortality in old critically ill COVID-19 patients: a multicenter prospective observational cohort study

**DOI:** 10.1007/s15010-023-02001-2

**Published:** 2023-03-01

**Authors:** Timo Mayerhöfer, Sebastian Klein, Bernhard Wernly, Hans Flaatten, Bertrand Guidet, Dylan W. De Lange, Jesper Fjølner, Susannah Leaver, Michael Beil, Sigal Sviri, Raphael Romano Bruno, Antonio Artigas, Peter Vernon van Heerden, Bernardo Bollen Pinto, Joerg C. Schefold, Rui Moreno, Maurizio Cecconi, Wojciech Szczeklik, Christian Jung, Michael Joannidis, COVIP study group, Philipp Eller, Dieter Mesotten, Pascal Reper, Sandra Oeyen, Walter Swinnen, Helene Brix, Jens Brushoej, Maja Villefrance, Helene Korvenius Nedergaard, Anders Thais Bjerregaard, Ida Riise Balleby, Kasper Andersen, Maria Aagaard Hansen, Stine Uhrenholt, Helle Bundgaard, Aliae A. R. Hussein Mohamed, Rehab Salah, Yasmin Khairy NasrEldin Mohamed Ali, Kyrillos Wassim, Yumna A Elgazzar, Samar Tharwat, Ahmed Y. Azzam, Ayman abdelmawgoad Habib, Hazem Maarouf Abosheaishaa, Mohammed A Azab, Arnaud Galbois, Cyril Charron, Emmanuel Guerot, Guillaume Besch, Jean-Philippe Rigaud, Julien Maizel, Michel Djibré, Philippe Burtin, Pierre Garcon, Saad Nseir, Xavier Valette, Nica Alexandru, Nathalie Marin, Marie Vaissiere, Gaëtan Plantefeve, Thierry Vanderlinden, Igor Jurcisin, Buno Megarbane, Anais Caillard, Arnaud Valent, Marc Garnier, Sebastien Besset, Johanna Oziel, Jean-herlé Raphaelen, Stéphane Dauger, Guillaume Dumas, Bruno Goncalves, Gaël Piton, Malte Kelm, Georg Wolff, Eberhard Barth, Ulrich Goebel, Eberhard Barth, Anselm Kunstein, Michael Schuster, Martin Welte, Matthias Lutz, Patrick Meybohm, Stephan Steiner, Tudor Poerner, Hendrik Haake, Stefan Schaller, Detlef Kindgen-Milles, Christian Meyer, Muhammed Kurt, Karl Friedrich Kuhn, Winfried Randerath, Jakob Wollborn, Zouhir Dindane, Hans-Joachim Kabitz, Ingo Voigt, Gonxhe Shala, Andreas Faltlhauser, Nikoletta Rovina, Zoi Aidoni, Evangelia Chrisanthopoulou, Antonios Papadogoulas, Mohan Gurjar, Ata Mahmoodpoor, Abdullah khudhur Ahmed, Brian Marsh, Ahmed Elsaka, Vittoria Comellini, Ahmed Rabha, Hazem Ahmed, Silvio a Namendys-Silva, Abdelilah Ghannam, Martijn Groenendijk, Marieke Zegers, Dylan de Lange, Alex Cornet, Mirjam Evers, Lenneke Haas, Tom Dormans, Willem Dieperink, Luis Romundstad, Britt Sjøbø, Finn H Andersen, Hans Frank Strietzel, Theresa Olasveengen, Michael Hahn, Miroslaw Czuczwar, Ryszard Gawda, Jakub Klimkiewicz, Maria de Lurdes Campos Santos, André Gordinho, Henrique Santos, Rui Assis, Ana Isabel Pinho Oliveira, Mohamed Raafat Badawy, David Perez-Torres, Gemma Gomà, Mercedes Ibarz Villamayor, Angela Prado Mira, Patricia Jimeno Cubero, Susana Arias Rivera, Teresa Tomasa, David Iglesias, Eric Mayor Vázquez, Cesar Aldecoa, Aida Fernández Ferreira, Begoña Zalba-Etayo, Isabel Canas-Perez, Luis Tamayo-Lomas, Cristina Diaz-Rodriguez, Susana Sancho, Jesús Priego, Enas M.Y. Abualqumboz, Momin Majed Yousuf Hilles, Mahmoud Saleh, Nawfel Ben-HAmouda, Andrea Roberti, Alexander Dullenkopf, Yvan Fleury, Joerg C Schefold, Mohammed Al-Sadawi, Nicolas Serck, Elisabeth Dewaele, Pritpal Kumar, Camilla Bundesen, Richard Innes, James Gooch, Lenka Cagova, Elizabeth Potter, Michael Reay, Miriam Davey, Sally Humphreys, Caroline Hauw Berlemont, Benjamin Glenn Chousterman, François Dépret, Alexis Ferre, Lucie Vettoretti, Didier Thevenin, Andreas Faltlhauser, Milena Milovanovic, Philipp Simon, Marco Lorenz, Sandra Emily Stoll, Simon Dubler, Kristina Fuest, Francesk Mulita, Eumorifa Kondili, Ioannis Andrianopoulos, Iwan Meynaar, Alexander Daniel Cornet, Britt Sjøbøe, Anna Kluzik, Paweł Zatorski, Tomasz Drygalski, Joanna Solek-pastuszka, Dariusz Onichimowski, Jan Stefaniak, Karina Stefanska-Wronka, Ewa Zabul, Filipe Sousa Cardoso, Maria José Arche Banzo, Teresa Maria Tomasa-Irriguible, Ángela Prado Mira, Susana Arias-Rivera, Fernando Frutos-Vivar, Sonia Lopez-Cuenca, Pablo Ruiz de Gopegui, Nour Abidi, Ivan Chau, Richard Pugh, Sara Smuts, Klemens Zotter

**Affiliations:** 1 Division of Intensive Care and Emergency Medicine, Department of Internal Medicine, Medical University Innsbruck, Anichstrasse 35, 6020 Innsbruck, Austria; 2 Department of Internal Medicine, General Hospital Oberndorf, Teaching Hospital of the Paracelsus Medical University Salzburg, Oberndorf, Salzburg, Austria; 3 Center for Public Health and Healthcare Research, Paracelsus Medical University of Salzburg, Salzburg, Austria; 4 Department of Anaesthesia and Intensive Care, Haukeland University Hospital, Bergen, Norway; 5 Assistance Publique, Hôpitaux de Paris, Sorbonne Universités, UPMC Univ Paris 06, INSERM, UMR_S 1136, Institut Pierre Louis d’Epidémiologie et de Santé Publique, Equipe: Epidémiologie Hospitalière Qualité et Organisation des Soins, 75012 Paris, France; 6 Department of Intensive Care Medicine, University Medical Center, University Utrecht, Utrecht, The Netherlands; 7 Department of Anaesthesia and Intensive Care, Viborg Regional Hospital, Viborg, Denmark; 8 Department of Critical Care, St George’s Hospital, London, UK; 9 Department of Medical Intensive Care, Hadassah Medical Center and Faculty of Medicine, Hebrew University of Jerusalem, Jerusalem, Israel; 10 Department of Cardiology, Pulmonology and Vascular Medicine, Medical Faculty, Heinrich-Heine-University Duesseldorf, Moorenstraße 5, 40225 Duesseldorf, Germany; 11 Intensive Intensive Care Medicine Department Corporacion Sanitària Parc Tauli CIBER Enfermedades Respiratorias Institut de Investigacio e Innovació I3PT, Autonomous University of Barcelona Sabadell, Sabadell, Spain; 12 Department of Anesthesia, Intensive Care and Pain Medicine, Hadassah Medical Center and Faculty of Medicine, Hebrew University of Jerusalem, Jerusalem, Israel; 13 Department of Anaesthesiology, Pharmacology and Intensive Care, Geneva University Hospitals, Geneva, Switzerland; 14 Department of Intensive Care Medicine, Inselspital, Bern University Hospital, University of Bern, Bern, Switzerland; 15 Unidade de Cuidados Intensivos Neurocríticos e Trauma, Hospital de São José, Centro Hospitalar Universitário de Lisboa Central, Faculdade de Ciências Médicas de Lisboa (Nova Médical School), Lisbon, Portugal; 16 Department of Anesthesia and Intensive Care Medicine, Humanitas Clinical and Research Center, IRCCS, Rozzano, MI Italy; 17 Department of Biomedical Sciences, Humanitas University, Rozzano, MI Italy; 18 Center for Intensive Care and Perioperative Medicine, Jagiellonian University Medical College, Kraków, Poland

**Keywords:** SARS-CoV-2, Intensive care unit, Elderly, Old, Critical care, Ventilation, Risk factors

## Abstract

**Background:**

Several studies have found an association between diabetes mellitus, disease severity and outcome in COVID-19 patients. Old critically ill patients are particularly at risk. This study aimed to investigate the impact of diabetes mellitus on 90-day mortality in a high-risk cohort of critically ill patients over 70 years of age.

**Methods:**

This multicentre international prospective cohort study was performed in 151 ICUs across 26 countries. We included patients ≥ 70 years of age with a confirmed SARS-CoV-2 infection admitted to the intensive care unit from 19th March 2020 through 15th July 2021. Patients were categorized into two groups according to the presence of diabetes mellitus. Primary outcome was 90-day mortality. Kaplan–Meier overall survival curves until day 90 were analysed and compared using the log-rank test. Mixed-effect Weibull regression models were computed to investigate the influence of diabetes mellitus on 90-day mortality.

**Results:**

This study included 3420 patients with a median age of 76 years were included. Among these, 37.3% (*n* = 1277) had a history of diabetes mellitus. Patients with diabetes showed higher rates of frailty (32% vs. 18%) and several comorbidities including chronic heart failure (20% vs. 11%), hypertension (79% vs. 59%) and chronic kidney disease (25% vs. 11%), but not of pulmonary comorbidities (22% vs. 22%). The 90-day mortality was significantly higher in patients with diabetes than those without diabetes (64% vs. 56%, *p* < 0.001). The association of diabetes and 90-day mortality remained significant (HR 1.18 [1.06–1.31], *p* = 0.003) after adjustment for age, sex, SOFA-score and other comorbidities in a Weibull regression analysis.

**Conclusion:**

Diabetes mellitus was a relevant risk factor for 90-day mortality in old critically ill patients with COVID-19.

**Study registration:**

NCT04321265, registered March 19th, 2020.

**Supplementary Information:**

The online version contains supplementary material available at 10.1007/s15010-023-02001-2.

## Background

SARS-CoV-2 leads to various manifestations, ranging from asymptomatic infections, to mild symptoms or severe illness and even death [1]. During the COVID-19 pandemic intensive care units were heavily affected worldwide, and several studies have investigated risk factors for a severity and a negative outcome [2, 3]. In addition to well-established vaccinations against COVID-19 [4, 5], antiviral drugs and monoclonal antibodies are now available to prevent a severe course of COVID-19 [6–8]. However, it is still important to identify risk factors associated with a worse outcome.

Age and frailty appear to be important predictors of critical illness and mortality in patients infected with SARS-CoV-2 [9, 10]. Furthermore, various comorbidities including obesity, chronic kidney disease (CKD) and diabetes mellitus (DM) have also been identified as significant risk factors [10].

Multiple studies have shown that patients with type 2 DM have an increased risk for hospitalisation, severe disease, intensive care unit (ICU) admission, longer length of stay, and mortality in COVID-19 [10–14]. Meta-analyses confirmed an independent association between DM with a more than two-fold increased risk for severe disease and mortality [15, 16]. For DM, the first ICU cohort studies from Wuhan suggested a rate of around 20% of patients with known DM in ICUs [17]. Higher rates of about 30% have been reported in Europe and the USA [18–20]. Type 2 DM is more common in advanced age [21]. Concomitantly, the prevalence of other comorbidities also increases with age, and frailty also becomes more predominant. The specific impact of each risk factor is therefore difficult to assess. This is especially true for old critically ill patients, where other comorbidities and frailty are more prevalent than in younger individuals. The COVID-19 disease in Very Elderly Intensive care Patients (COVIP) study focuses on prognostic factors and outcome of old ICU patients (≥ 70 years) with COVID-19. Whether DM is also a relevant risk factor in this vulnerable cohort remains unclear.

Therefore, we aimed to investigate the influence of DM on outcomes in a cohort of already vulnerable old critically ill COVID-19 patients.

## Methods

### Study design, setting and participants

This study is a secondary analysis of the ongoing COVIP-trial (NCT04321265), a multicentre international observational prospective study that includes critically ill patients with COVID-19 over or equal to 70 years of age and is part of the Very old Intensive care Patients (VIP) project (www.vipstudy.org). A list of collaborators is shown in the Additional file 1. This study included participants from 151 ICUs across 26 countries in Europe, Asia, Africa and America. In all centres, ethical approval was required for participation and in most countries informed consent was obtained for enrollment in the study, in accordance with local regulations. This study was conducted in accordance with the Helsinki Declaration. The detailed methods of this study were published previously [22].

In brief, patients with a positive polymerase-chain-reaction for SARS-CoV-2, who were 70 years of age or older and admitted to an ICU were eligible for the study. For this analysis, we included all records in the database from 19th March 2020 through 15th July 2021. Data collection for each patient began on admission to the ICU. The day of admission was defined as the first day, and all subsequent days were numbered consecutively. For this analysis, all critically ill patients ≥ 70 years with known DM status (yes or no) were included (definitions of comorbidities and treatment limitations in additional file 2). We excluded 507 patients with unknown DM status.

### Data collection

All participating centres used a standardised online electronic case report form, that recorded baseline characteristics (Table 1), the sequential organ failure assessment (SOFA) score at admission, the need for non-invasive or invasive ventilation, prone positioning, tracheostomy, use of vasopressors and renal replacement therapy and any restriction of life-sustaining treatment during the ICU stay. The database ran on a secure server set up at Aarhus University, Denmark.

**Table 1:** Patient characteristics of the study population with and without diabetes mellitus

	No diabetes	Diabetes	*p-*value
	*n* = 2143	*n* = 1277	
Characteristics			
Sex			0.12
Male, % (*n*)	70% (1505)	68% (864)	
Female, % (*n*)	30% (638)	32% (413)	
Age at admission	76 (5)	76 (5)	0.91
Age at admission			0.70
Age < 80 years, % (*n*)	78% (1662)	78% (998)	
Age > 79 years, % (*n*)	22% (480)	22% (279)	
BMI	28 (5)	30 (6)	< 0.001
SOFA score on admission	5 (3)	6 (3)	< 0.001
Comorbidities			
Ischemic heart disease, % (*n*)	19% (405)	31% (393)	< 0.001
Chronic kidney disease, % (*n*)	11% (238)	25% (319)	< 0.001
Arterial hypertension, % (*n*)	59% (1263)	79% (1005)	< 0.001
Pulmonary comorbidity, % (*n*)	22% (459)	22% (274)	0.92
Chronic heart failure, % (*n*)	11% (241)	20% (249)	< 0.001
Clinical Frailty Scale Score	3 (2)	4 (2)	< 0.001
Presence of Frailty (CFS > 4)			< 0.001
No frailty, % (*n*)	82% (1604)	68% (767)	
Frailty, % (*n*)	18% (345)	32% (363)	
Therapeutic measures			
Intubation and mechanical ventilation, % (*n*)	71% (1512)	67% (854)	0.024
Non-invasive ventilation, % (*n*)	25% (535)	30% (375)	0.003
Tracheostomy, % (*n*)	19% (399)	15% (185)	0.002
Vasoactive drugs, % (*n*)	67% (1431)	63% (789)	0.004
Corticosteroids, % (*n*)	68% (1460)	74% (944)	0.003
Renal Replacement Therapy, % (*n*)	13% (286)	18% (224)	< 0.001
Treatment limitations			
Any treatment limitation, % (*n*)	36% (772)	32% (398)	0.006
Life sustaining care withheld, % (*n*)	29% (625)	28% (347)	0.25
Life sustaining care withdrawn, % (*n*)	20% (426)	17% (210)	0.015

*CFS* clinical frailty scale

### Outcomes

The primary outcome was 90-day mortality, the secondary outcomes were overall survival to ICU discharge, survival at 30 days after ICU admission and ICU length of stay.

### Statistical analysis

Kolmogorov–Smirnov test was used to test for normal distribution. Differences between the two groups were calculated using the Mann–Whitney *U* test and Chi-squared test for not normally distributed data and Student’s *t*-test for normally distributed data. The study population was divided into patients with and without DM.

Mixed-effects Weibull regression analysis was applied with the center as a random effect and DM as fixed effects to evaluate DM as a possible independent predictor for 90-day mortality. For a better estimation of the impact of DM on outcome three different models were created (Model A, B and C). Model A included age, sex and the SOFA score, to account for disease severity. In Model B frailty was added as a recently established risk factor for mortality in COVID-19 patients [22], and in Model C, all other comorbidities obtained in the COVIP study according to the protocol were added as additional variables. Adjusted hazard ratios with corresponding 95% confidence intervals (95% CI) were calculated. Furthermore, 90-day mortality was analysed using Kaplan–Meier curves for patients with and without DM. Differences were assessed using the log-rank test.

To further investigate the influence of DM on 90-day mortality in certain subgroups, we created a forest plot depicting univariable hazard ratios for patients with DM in each subgroup.

All tests were two-sided, and a *p* value of < 0.05 was considered statistically significant. Some patients were excluded for subgroup analyses due to missing values. For this reason, not all patient numbers add up to 100%.

Stata 17 was used for all statistical calculations (StataCorp LLC, 4905 Lakeway Drive, College Station, Brownsville, Texas, USA).

## Results

### Patients

In total, 3420 critically ill patients aged ≥ 70 years from the COVIP study were included in this analysis. Baseline characteristics are presented in detail in Table 1. A pre-existing diagnosis of DM was present in 1277 (37.3%) patients. The median age of the total cohort was 76 years and similar in both groups (p = 0.91). The same applies to sex distribution: among patients with and without DM 70% were men and 30% women.

### Comorbidities

Patients with DM had a higher median BMI (30 kg/m^2^ vs. 28 kg/m^2^, *p* < 0.001) and a higher SOFA score at ICU admission (6 vs 5, *p* < 0.001), compared to patients without the diagnosis. The most common comorbidities such as ischemic heart disease, CKD, hypertension and chronic heart failure (CHF) were significantly more frequent in patients with DM. Furthermore, these patients had a higher median score on the clinical frailty scale and a higher proportion was known to be frail (32% vs. 18%, *p* < 0.001). No difference was observed in the frequency of pulmonary comorbidities.

### Therapeutic measures during ICU stay

In 71% (n = 1512) of patients without DM and in 67% (*n* = 854) of patients with DM MV was used (*p* = 0.024). Tracheostomy was performed more frequently in patients without DM (19% vs. 15%, *p* = 0.002), as was the use of vasoactive drugs (67% vs. 63%, *p* = 0.004). By contrast, renal replacement therapy was used significantly more often in patients with DM (18% vs. 13%, *p* < 0.001).

Treatment limitations were less common in patients with DM (32% vs 36%, *p* = 0.006, Table 2) and the ICU length of stay was not significantly different in patients with or without DM.

**Table 2:** Outcome of patients with and without diabetes mellitus

	No diabetes	diabetes	*p-*value
	*n* = 2143	*n* = 1277	
Outcome			
Mortality at 30 days, % (*n*)	51% (1092)	59% (751)	< 0.001
Mortality at 90 days, % (*n*)	56% (1194)	64% (811)	< 0.001
Mortality at ICU discharge, % (*n*)	49% (1037)	55% (699)	< 0.001
ICU length of stay (h)	512 (1188)	455 (1297)	0.19

*ICU* Intensive Care Unit

### Outcome

**Fig. 1:**
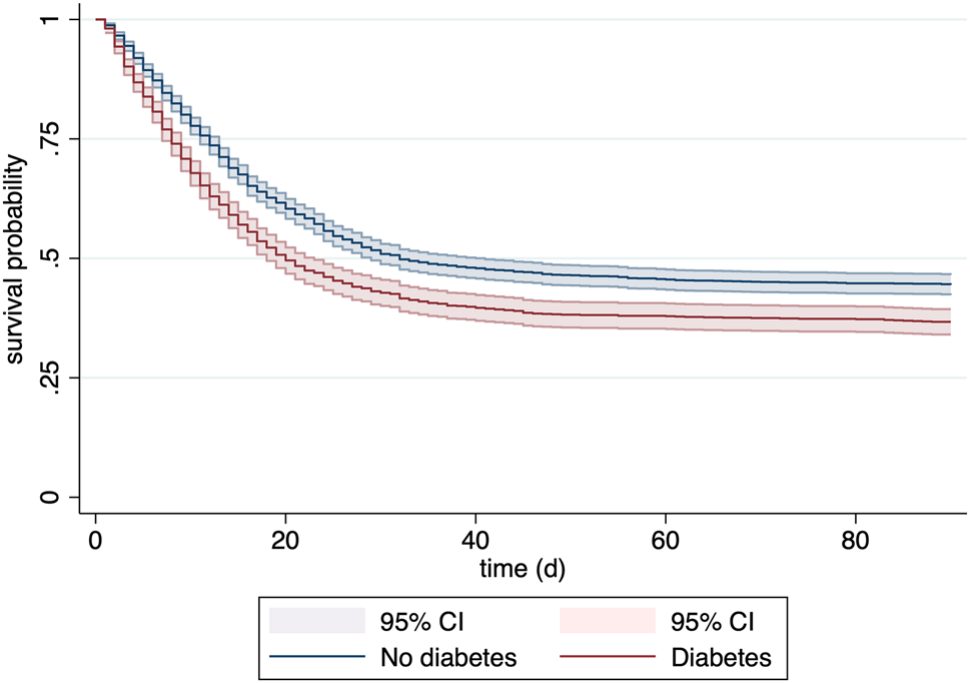
Kaplan–Meier survival analysis displaying the survival probability in relation to the diabetes mellitus status at admission. *CI* confidence interval

The 90-day mortality was significantly higher in patients with DM than in patients without DM (Table 2). The same applies to ICU and 30-day mortality. The ICU length of stay was not significantly different in patients with or without DM. As illustrated by the Kaplan–Meier analysis, DM was significantly associated with an impaired survival probability (Fig. 1).

In the univariable analysis, DM mellitus was a significant predictor of 90-day mortality with a hazard ratio of 1.29 (95% CI 1.18–1.41, *p* < 0.001).

To evaluate the relevance of DM in the prediction of 90-day mortality in very old critically ill patients, we created three different models (see Table 3). The association between DM and 90-day mortality was also significant after adjustment for age, sex and the SOFA score in Model A. When frailty or other comorbidities were added to the model (Model B and Model C), DM remained a significant but weaker predictor for mortality.

**Table 3:** Mixed-effects Weibull proportional hazard regression analysis for prediction of 90-day mortality

	Model A		Model B		Model C	
	HR (95% CI)	*p* value	HR (95% CI)	*p *value	HR (95% CI)	*p* value
Diabetes	1.24 (1.14–1.38)	< 0.001	1.17 (1.06–1.30)	0.002	1.18 (1.06–1.31)	0.003
Age	1.06 (1.05–1.73)	< 0.001	1.06 (1.04–1.07)	< 0.001	1.06 (1.04–1.06)	< 0.001
Sex	0.97 (0.88–1.08)	0.582	0.92 (0.83–1.03)	0.155	0.94 (0.84–1.05)	0.268
SOFA Score	1.14 (1.13–1.16)	< 0.001	1.13 (1.11–1.15)	< 0.001	1.13 (1.11–1.15)	< 0.001
Frailty			1.82 (1.60–2.06)	< 0.001	1.69 (1.48–1.93)	< 0.001
Ischemic heart disease					1.06 (0.93–1.20)	0.387
Chronic kidney disease					1.33 (1.16–1.52)	< 0.001
Arterial hypertension					0.85 (0.76–0.95)	0.005
Pulmonary comorbidity					1.11 (0.99–1.27)	0.071
Chronic heart failure					1.09 (0.93–1.27)	0.296

*HR* hazard ratio, *CI* confidence interval, *SOFA* Sequential Organ Failure Assessment, Center as random effect and Diabetes as fixed effects

**Fig. 2:**
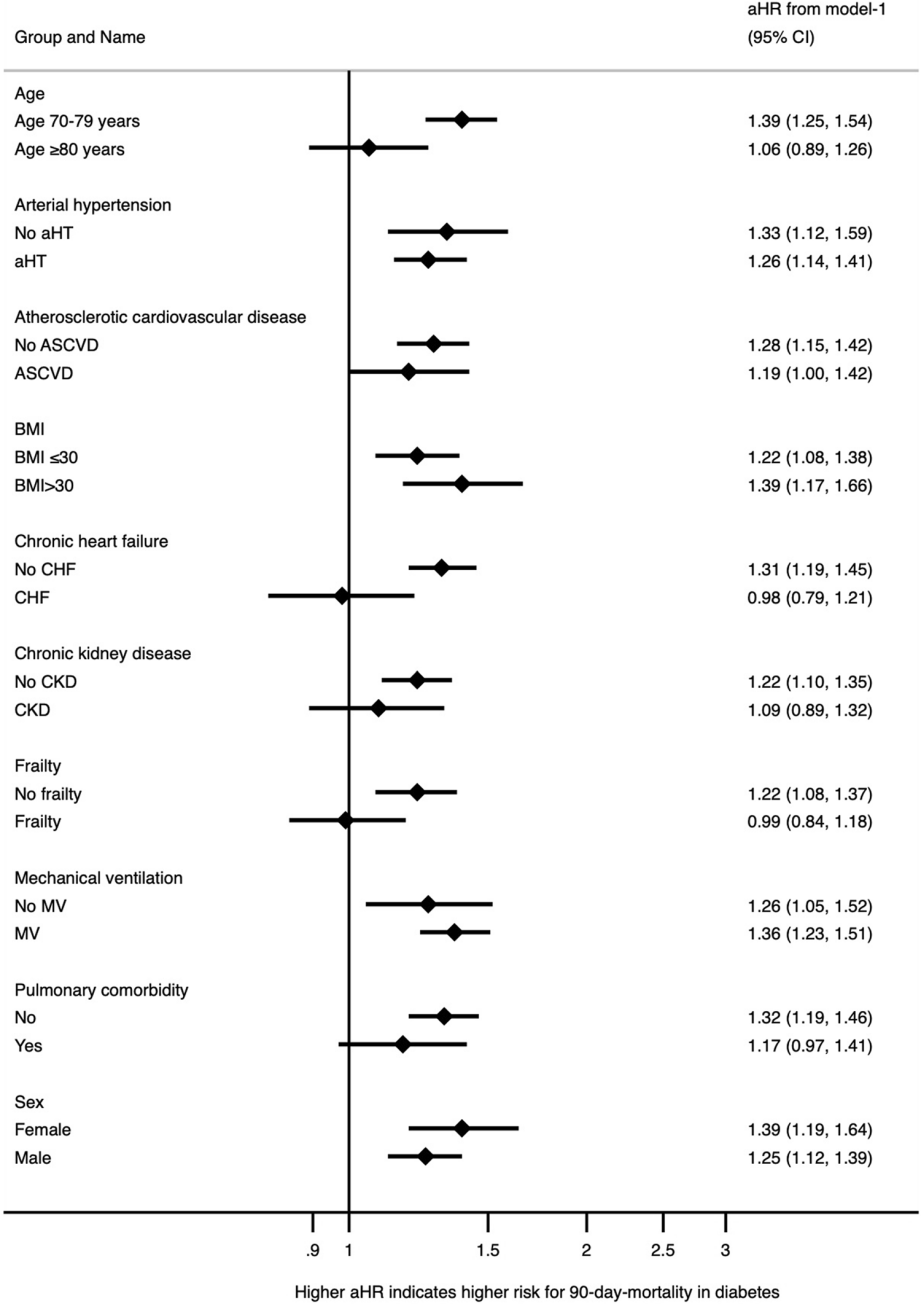
Forest plot for univariable hazard ratios for patients with DM in different subgroups. *CI* confidence interval, *aHR* adjusted hazard ratio, *aHT* arterial hypertension, *ASCVD* atherosclerotic cardiovascular disease, *BMI* body mass index, *CHF* chronic heart failure, *CKD* chronic kidney disease, *MV* mechanical ventilation

The univariate hazard ratio for DM was higher in the subgroup of patients without frailty, without CHF and between the age of 70 and 79. However, for patients with frailty, with CHF or over 80 years of age, no association was found between 90d mortality and the presence of DM (Fig. 2).

## Discussion

In this large multicentre cohort study of old critically ill COVID-19 patients, we observed a high rate of DM, with an independent association with 90-day mortality. The effect was more pronounced in younger individuals (70–79 years) and in patients without frailty, CKD and CHF. However, patients with DM had a significantly higher rate of comorbidities. These comorbidities are usually considered to be a consequence of diabetes-related end-organ damage.

Understanding the most important factors for an unfavorable outcome in critically ill COVID-19 patients is crucial. Older individuals are more commonly affected by a severe course of disease [23–26]. However, age is not suitable as the sole factor for predicting outcome in critically ill COVID-19 patients. Therefore, knowledge about the impact of other comorbidities is of great importance, especially in an old population.

Several studies have shown that COVID-19 patients with DM have a worse outcome than patients without DM. In an early meta-analysis of 33 studies, Kumar et al*.* found a more than two-fold increased risk of mortality and an increased severity of disease in COVID-19 patients with DM when compared to those without DM [15]. Since DM is often associated with multiple other comorbidities, it remained unclear whether this association was independent of other factors. A recently published national cohort study from England showed that DM is indeed an independent predictor for mortality [27]. However, the transferability of results from a national study to other countries may be limited.

DM was one of the most frequent comorbidities in our population at 37%. Compared to other cohort studies of critically ill patients, this number is slightly higher [18, 23, 28, 29]. Our study refers only to old individuals over 70 years of age, in whom DM generally has a greater prevalence than in the overall population [21]. In support of our data, a cohort study from Germany with a comparatively old study population yielded similar numbers [25].

Our multicentre study now provides evidence that even in a highly vulnerable cohort of old critically ill patients the presence of DM is associated with higher 90-day mortality. Even when adjusting for age, frailty and other comorbidities, DM remained independently associated with mortality. However, since age and frailty are such important characteristics [23, 30], the influence of DM was reduced.

The impact of DM was more prominent in younger patients in our cohort (70–79 years). A retrospective study from Mexico and another study from France made similar observations, describing a decrease in the association of DM and death with increasing age, although their cohorts started at a much younger age [31, 32]. For patients age 80 years and above the influence of DM seems to be reduced in relation to other comorbidities. When comparing the prevalence of comorbidities in our cohort, several diseases such as CKD, cardiovascular disease and CHF showed rates which were between 1.5 and 2 times higher for patients with DM compared to those without. CKD and CHF are both risk factors for a severe course of COVID-19 [10]. Since these diseases are considered typical manifestations of end-organ damage due to DM they cannot be seen as factors independent of DM. The same is true for frailty which was roughly twice as frequent in our critically ill patients with DM. Frailty has been proven to be a relevant prognostic marker for outcomes in critically ill patients with COVID-19 [22] as well as without COVID-19 [33].

Thus, we may infer, that although frailty and other comorbidities seem to mask the influence of DM in older patients, they might also be a consequence of DM itself and therefore be interrelated with this underlying disease.

As recently reported treatment limitations were more frequent in old COVID-19 patients compared to patients without COVID-19 disease [34]. Varying rates of treatment limitations in patients with and without DM might have the potential to affect the outcome. However, in our study, treatment limitations were even less common in patients with DM. Therefore, they cannot be responsible for the higher mortality observed in this group.

In addition, DM, especially type 2 DM, is further associated with obesity [35]. In our study patients with DM had a higher body mass index than patients without DM. Obesity and DM negatively affect the outcome during infectious diseases by altering the immune response, which explains the poor outcome in these patients [36].

The main strengths of this study are its multicentre study design and a large number of patients included. However, some limitations such as the observational design of this study must be considered when interpreting the results. First, some important values are missing such as blood glucose levels or HbA1c. These values might provide additional information especially on glycemic control, which was associated with outcome in COVID-19 patients in another study [37]. Second, we did not differentiate between type 1 and type 2 DM. Since type 2 DM is much more prevalent in older individuals [21] and patients with type 1 DM have a reduced life expectancy [38], it is likely that nearly all patients with DM in our study cohort had type 2 DM. However, it cannot be completely excluded, that some patients with type 1 DM influenced the results. Moreover, there is a small risk of selection bias, since 507 patients were excluded due to missing DM status.

## Conclusion

DM is independently associated with an impaired outcome in old critically ill COVID-19 patients. The influence of DM was strongest in “younger” patients without frailty. DM might be of additional value for risk prediction even in old critically ill COVID-19 patients who are already at high risk of a poor outcome.

## Supplementary Information

Below is the link to the electronic supplementary material.

Supplementary file1 (DOCX 41 KB)

Supplementary file2 (DOCX 14 KB)

## Data Availability

Individual participant data that underlie the results reported in this article are available to investigators whose proposed use of the data has been approved by the COVIP steering committee.
