# Supplementary material for: Diabetes mellitus is associated with 90-day mortality in old critically ill COVID-19 patients: a multicenter prospective observational cohort study
Source: Infection. 2023 Mar 1;51(5):1407–15. doi: 10.1007/s15010-023-02001-2 (PMC9974396; doi:10.1007/s15010-023-02001-2)
Supplement: Supplementary file 2 — Supplementary file2 (DOCX 14 KB) [file 15010_2023_2001_MOESM2_ESM.docx]

Definition of comorbidities and treatment limitations:

Diabetes mellitus: documented evidence of diabetes mellitus or reported by the patient or their relatives. Prescription of anti-diabetic medication or insulin on the drug chart.

Ischaemic heart disease: documented abnormal coronary angiography, known coronary artery disease, previous percutaneous coronary intervention (PCI) or coronary bypass surgery

Chronic renal failure: documented evidence of chronic renal insufficiency Grade 3 or higher, creatinine clearance <60ml/min or chronic dialysis

Arterial hypertension: documented evidence of any grade of chronic arterial hypertension or prescription of anti-hypertensive medication.

Pulmonary disease: documented evidence of or medication prescribed for chronic pulmonary disease of any aetiology (bronchial asthma, COPD, pulmonary fibrosis), or clinical or radiological signs of chronic pulmonary disease

Chronic heart failure: documented evidence of or medication prescribed for chronic heart failure of any aetiology or echocardiographic or radiological signs of chronic heart failure.

Treatment limitations: Withholding treatment is defined as the decision not to start or escalate a life-sustaining intervention, such as not to perform CPR if a patient had a cardiac arrest or to decide not to treat, with renal replacement therapy. Withdrawing treatment is defined as a decision to stop a life-sustaining intervention presently being given, such as stopping a norepinephrine infusion knowing that the patient may not survive without the treatment.
